# Xanthine Oxidase-Derived ROS Upregulate Egr-1 via ERK1/2 in PA Smooth Muscle Cells; Model to Test Impact of Extracellular ROS in Chronic Hypoxia

**DOI:** 10.1371/journal.pone.0027531

**Published:** 2011-11-28

**Authors:** Tanya Hartney, Rahul Birari, Sujatha Venkataraman, Leah Villegas, Maylyn Martinez, Stephen M. Black, Kurt R. Stenmark, Eva Nozik-Grayck

**Affiliations:** 1 Department of Pediatrics, University of Colorado Denver, Aurora, Colorado, United States of America; 2 Cardiovascular Pulmonary Research Laboratory, University of Colorado Denver, Aurora, Colorado, United States of America; 3 Georgia Health Sciences University, Augusta, Georgia, United States of America; University of Giessen Lung Center, Germany

## Abstract

Exposure of newborn calves to chronic hypoxia causes pulmonary artery (PA) hypertension and remodeling. Previous studies showed that the redox-sensitive transcription factor, early growth response-1 (Egr-1), is upregulated in the PA of chronically hypoxic calves and regulates cell proliferation. Furthermore, we established in mice a correlation between hypoxic induction of Egr-1 and reduced activity of extracellular superoxide dismutase (EC-SOD), an antioxidant that scavenges extracellular superoxide. We now hypothesize that loss of EC-SOD in chronically hypoxic calves leads to extracellular superoxide-mediated upregulation of Egr-1. To validate our hypothesis and identify the signaling pathways involved, we utilized PA tissue from normoxic and chronically hypoxic calves and cultured calf and human PA smooth muscle cells (PASMC). Total SOD activity was low in the PA tissue, and only the extracellular SOD component decreased with hypoxia. PA tissue of hypoxic calves showed increased oxidative stress and increased Egr-1 mRNA. To mimic the *in vivo* hypoxia-induced extracellular oxidant imbalance, cultured calf PASMC were treated with xanthine oxidase (XO), which generates extracellular superoxide and hydrogen peroxide. We found that 1) XO increased Egr-1 mRNA and protein, 2) XO induced the phosphorylation of ERK1/2 and, 3) pretreatment with an ERK1/2 inhibitor prevented induction of Egr-1 by XO. siRNA knock-down of EC-SOD in human PASMC also upregulated Egr-1 mRNA and protein, activated ERK1/2, and enhanced SMC proliferation and reduced apoptosis. We conclude that an oxidant/antioxidant imbalance arising from loss of EC-SOD in the PA with chronic hypoxia induces Egr-1 via activation of ERK1/2 and contributes to pulmonary vascular remodeling.

## Introduction

Infants, children and adults with chronic lung diseases complicated by alveolar hypoxia are at risk for developing pulmonary hypertension, which is associated with a high morbidity and mortality [1]. Exposure of animals to chronic hypoxia is a well-established and useful model to interrogate the mechanisms that may contribute to human disease. Accumulating evidence indicates that reactive oxygen species including superoxide (O_2_^.−^) are important in the pathogenesis of pulmonary hypertension, including chronic hypoxia-induced pulmonary hypertension [2], [3]. There are a number of known sources of O_2_^.−^ in the pulmonary artery including NADPH oxidase, the mitochondrial electron transport chain, uncoupled endothelial nitric oxide synthase and xanthine oxidase (XO) that have been implicated in generation of O_2_^.−^ in response to hypoxia [3], [4]. There is accumulating evidence that O_2_^.−^ generated specifically in the extracellular compartment contributes to the development of pulmonary hypertension. The antioxidant enzyme which defends against extracellular O_2_^.−^, extracellular superoxide dismutase (EC-SOD or SOD3), is highly expressed in the pulmonary circulation, and its level of expression may modulate the development of pulmonary hypertension. Lung EC-SOD expression and activity decreases in rodent models associated with oxidative stress, including hypoxia and bleomycin-induced lung injury, as well as in the bronchus of humans with end-stage idiopathic pulmonary arterial hypertension [5]–[9]. Furthermore, enhancing lung EC-SOD activity either in genetically engineered mice or with adenoviral gene delivery protects against pulmonary hypertension and pulmonary vascular remodeling due to monocrotaline, bleomycin, or chronic hypoxia [10]–[12]. Overexpression of EC-SOD protects by limiting fibrosis and inflammation and prevents the upregulation of key genes involved in these processes. Among many redox-regulated genes, the transcription factor, early growth response-1 (Egr-1) is of interest because we and others have shown it increases in the lung and pulmonary vascular cells early in response to hypoxia and activates a number of downstream targets critical to proliferation, fibrosis and inflammation [10], [11], [13]–[19]. Therefore, Egr-1 can play a critical role in pulmonary vascular remodeling though its regulation in pulmonary hypertension by ROS is not clearly understood.

The contribution of EC-SOD to the pathogenesis of neonatal pulmonary hypertension has not been substantially investigated. Broadly it has been recognized that the neonatal lung is susceptible to oxidative stress due to the developmental regulation of antioxidant defenses [20]. The neonatal calf is particularly susceptible to hypoxia-induced pulmonary hypertension, with severe inflammation, pulmonary vascular remodeling and pulmonary hypertension, and Egr-1 is increased in the pulmonary artery in the chronically hypoxic neonatal calf [19]. The calf model is a useful model not only because of the severe pathology similar to human disease, but also because of the availability of primary pulmonary artery cells from the neonatal calf to test mechanisms responsible for the pathogenesis of pulmonary hypertension. We hypothesized that loss of EC-SOD specifically within the pulmonary artery of the newborn calf in response to hypoxia leads to extracellular superoxide-mediated upregulation of Egr-1. We utilized PA tissue isolated from normoxic and chronically hypoxic calves along with cultured calf to test this hypothesis and investigate the signaling pathway involved in the ROS modulation of Egr-1. In addition, we also knocked-down EC-SOD in human PASMC to directly test the contribution of EC-SOD to Egr-1 expression as well as SMC proliferative ability.

## Methods

### Animal Model and Tissue Harvesting

This study was carried out in strict accordance with the recommendations in the Guide for the Care and Use of Laboratory Animals of the National Institutes of Health. All animal studies were approved by the Institutional Animal Use and Care Committees (Colorado State University School of Veterinary Medicine, Fort Collins, CO (Protocol 10-1927A) or University of Colorado School of Medicine, Aurora, CO (Protocol # 71108(03)1E). Chronic exposure to hypoxia is a well-established model of pulmonary hypertension, characterized by elevated pulmonary artery pressures and pulmonary artery remodeling. In this study, 1-day-old male Holstein calves (Laluna Dairy Farm, Fort Collins) were placed in a hypobaric hypoxia chamber at P_B_ 445 mm Hg (simulating 15,000 ft or 12% FIO_2_) for 14 days along with age-matched calves maintained in ambient Denver altitude (P_B_ 640 mm Hg). Tissue from chronically hypoxic calves was harvested within the chamber under hypobaric hypoxic conditions while tissue from normoxic calves was obtained in normobaric atmosphere. Calves were euthanized with an overdose of pentobarbital sodium (160 mg/kg body weight) and lung tissue and intraparenchymal proximal pulmonary artery tissue containing all three layers of the vessel wall were rapidly dissected and flash frozen. Another pulmonary artery segment was placed in cold media for preparation of primary vascular cell lines. Tissue was flash frozen for analysis.

### Cell Culture model

To test how extracellular O_2_^.−^ generated in the pulmonary artery wall can upregulate the redox sensitive transcription factor, Egr-1, experiments were done with smooth muscle cells (SMC) isolated from the pulmonary artery of normoxic or chronically hypoxic calves using an explant technique as previously described [21]. This cell type was selected as a key cell type implicated in the pathogenesis of pulmonary hypertension that also expresses Egr-1 [19], [22]. Cells were grown in DMEM with 10% bovine calf serum (Gemini, West Sacramento, CA) and supplemented with Cellgro 1% NEAA (Mediatech, Inc, Manassas, VA), 4 mM Cellgro L-Glutamine (Mediatech, Inc) and Cellgro pen-strep (100 I.U/mL pen and 100 µg/mL strep, Mediatech, Inc) and maintained in humidified incubator at 5% CO_2_ and 37°C. Cells were used between passages 4 and 8. The cell culture data, unless specified otherwise in a particular experiment, is derived from three separate wells or plates of cells derived from a single chronically hypoxic calf and performed on a single day to control for day-to-day variability and enable comparisons between experimental groups. The experiments were repeated on at least 3 different days with cells from at least 3 different calves to ensure reproducibility. The qPCR was also performed in triplicate for each cell isolation. Human PASMC purchased from Lonza were grown in designated SMC media (Clonetics LONZA smBm media cat no: CC3181).

### Treatment groups

Cells were treated with XO because it is an enzyme known to be upregulated in the pulmonary circulation in models of pulmonary hypertension and it is a reproducible method to generate extracellular O_2_^.−^ in order to study the impact of O_2_^.−^ released in this particular cell compartment. Cells were treated with XO dissolved in phosphate buffered saline (PBS) (Sigma, St Louis, MO) and hypoxanthine (HX) (Sigma) dissolved in 0.1 M sodium hydroxide (NaOH). The optimal dose of XO and HX was established with a dose and time response curve. Based on pilot results, all remaining studies were carried out using cells treated for 1 hour with 8 mU/mL XO and 0.5 mM HX (XO/HX). Control cells for XO/HX treatment received the vehicles alone (PBS and 0.1 M NaOH). For studies with extracellular antioxidant treatments, cells were pretreated for 30 minutes with 500 U/mL SOD (Sigma) and/or catalase 600 U/mL (Boehringer Mannheim, Mannheim, Germany). Catalase was first dialyzed against PBS for 24 hours in PBS and activity determined as previously described [23]. Catalase was stored at −20°C until used. For MAPK inhibitor studies, cells were pretreated for 30 minutes with the ERK1/2 Inhibitor PD98059 (10 µM, Cell Signaling Danvers, MA) followed by the 1 hour treatment with XO. One series of PASMC were exposed in sealed humidified gas chambers to 21% or 1% oxygen tension with 5% CO_2_ and balanced N_2_ for 4 and 24 hours, as previously described, to evaluate the relative impact of hypoxia on Egr-1 expression and extracellular O_2_^.−^ release compared to XO/HX-treated cells [10]–[12]. An additional series of cells were treated for 2 hours with low dose (0.25 µM) or high dose (250 µM) antimycin A, an inhibitor of the mitochondrial electron transport chain, to generate endogenous O_2_^.−^. Antimycin A was dissolved in DMSO and the appropriate vehicle was used for control conditions.

### siRNA transfection

An siRNA targeting EC-SOD and a non-targeting siRNA were transfected into human PASMC (Lonza Walkersville, Walkersville, MD) using the siPORT NeoFX transfection reagent (Ambion). Cells (12×10^5^) in a 6-well plate, were transfected with a final concentration of 5 nM of non-targeting (negative control, siNEG)) or siRNA targeting EC-SOD, (siEC-SOD). The manufacturer's suggested protocol of a reverse transfection was followed. Transfected cells were then harvested after 48 h for mRNA or protein analysis. After every transfection, the EC-SOD knockdown was confirmed by qPCR.

### mRNA isolation from calf PA and cultured cells

For pulmonary artery segments, the fibrous tissue was first pulverized using a mortar and pestle on liquid nitrogen to facilitate homogenization. Cells, 4×10^4^ per well, were seeded in a 6 well plate (Corning, Lowell MA) and allowed to grow for 48 hours prior to treatment. RNA was isolated from tissue using TRIzol (Invitrogen, Carlsbad, CA) and from cells using the RNeasy Plus kit (Qiagen, Germantown, MD) according to manufacturers' instructions. The concentration and purity of each total RNA sample was determined by the NanoDrop® ND-1000 spectrophotometer (NanoDrop, Wilmington, DE). The integrity of total RNA samples was examined by 2100 Bioanalyzer and the RNA 6000 Nano Kit (Agilent Technologies).

### mRNA analysis by real time RT-PCR (qPCR)

RNA (1 µg per reaction) was reverse transcribed with the Maxima First Strand cDNA synthesis kit (Fermentas International Inc, Glen Burnie, MD). PCR was performed on the MyiQ Detection System (Bio-Rad, Hercules, CA) with the RT^2^ Real-Time SYBR Green/Fluorescein PCR master mix (SABiosciences, Frederick, MD). Reactions were run in triplicate and results analyzed by the 2 (−delta delta CT) Method, normalizing the gene copy numbers to hypoxanthine-guanine phosphoribosyltransferase (HPRT). Data are expressed in figures as either actual copies of Egr-1/HPRT or, to more easily visualize fold change in certain experiments, Egr-1/HPRT normalized to the mean expression of the control group. Primers for calf tissue were designed with NCBI Primer-BLAST software. Primers used were Egr-1 forward: CCTTCAG TACCCACCTCCTG Egr-1 reverse: AGGGCTTCTGATCTGGTGTG. HPRT forward: CCAAAGATGGTCA AGGTTGC HPRT reverse: GGGCATATCCCACAACAAAC. Human Egr-1 and GAPDH primers were purchased from SABiosciences. For human EC-SOD mRNA analysis, Taqman EC-SOD primers were purchased along with the corresponding GAPDH primers and qPCR performed with Taqman master mix according to instructions (Applied Biosciences).

### Protein isolation

Pulverized lung or pulmonary artery tissue was homogenized in RIPA buffer (Sigma) containing protease (Sigma) and phosphatase (Thermo Scientific, Rockford, IL) inhibitors and centrifuged to remove cellular debris. For total cell lysates from cultured cells, 5.0×10^5^ cells were seeded in 100 mm dishes (Corning) and allowed to grow for 48 hours prior to treatment. After appropriate treatment, media was removed and plates placed on ice, cells washed 2× PBS and then lysed in RIPA buffer (Sigma) containing protease (Sigma) and phosphatase (Thermo Scientific) inhibitors. Cells were scraped and placed in −80°C overnight followed by sonification and centrifugation at 4°C degrees to collect the supernatant. Protein concentration was determined using the Pierce 660 nm protein assay reagent (Thermo Scientific). Nuclear protein was extracted from calf PASMC (1×10^6^ cells per condition) using NE-PER Nuclear and Cytoplasmic Extraction Reagents according to product instructions (Pierce Biotechnology, Rockford, IL).

### Western blot analysis

For Western blot analysis with the phosphoERK1/2 and total ERK1/2 antibodies, 20 µg human or calf total PASMC protein was loaded on a 4–12% Bis-Tris Gel (Invitrogen, Carlsbad, CA) and protein separated by gel electrophoresis. Proteins were transferred to PDVF membranes using the semi-dry method (Invitrogen). Blots were blocked with 5% milk in Tris Buffered Saline with 0.05% Tween 20 (TBST) and probed with the primary monoclonal mouse phospho-p44/42 MAPK antibody (Cell Signaling, 1∶1000) overnight at 4°C followed by an anti-mouse HRP-conjugated secondary antibody (Millepore, Billerica, MA). Blots were developed with Enhanced Chemiluminescence (ECL) plus (Thermo Scientific) and then stripped with Restore Plus Western Blot Stripping Buffer according to kit instructions (Thermo Scientific) and reprobed with rabbit monoclonal Total ERK antibody (Cell Signaling, 1∶1000), for normalization: Bands were quantified by densitometry and expressed as the ratio of phosphoERK/totalERK. Blots from human PASMC total cell lysates were also probed with polyclonal rabbit anti-Egr-1 (Cell Signaling, 1∶500), cyclin D1 (Cell Signaling , 1∶1,000) and ß-actin as a loading control. For calf PASMC, to reduce non-specific binding by the Egr-1 antibody, likely due to poor cross-reactivity with the anti-human Egr-1 antibody, 25 µg nuclear extracts were used instead for Western blot. Equal nuclear protein loading was confirmed by Ponceau S staining (Millipore). For the Western blot with protein from pulmonary artery or lung tissue, 20–100 mg of protein homogenate was loaded on the gel as well as 1 mg purified bovine EC-SOD protein as a positive control. The blot was probed overnight at 4°C with 1∶1,000 rabbit anti-human EC-SOD ab in 5% milk, which recognizes bovine EC-SOD (purified EC-SOD and EC-SOD antibody kindly provided by Tim Oury, MD, PhD, University of Pittsburgh). Western blots for PA protein were also probed with antibodies against Cu,Zn SOD (Abcam, 1∶2,000) Mn SOD (Millipore, 1∶1,000), and ß-actin in protein homogenates from normoxic and chronically hypoxic calves.

### Reactive oxygen species measurements

2,3-bis-(2-methoxy-4-nitro-5-sulfophenyl)-2H-tetrazolium-5-carboxanilide, disodium salt (XTT) (Sigma) was used to detect extracellular O_2_^.−^ generated in response to XO/HX or hypoxia in cultured calf PASMC. SMC (3×10^4^), were plated in triplicate in a 96 well plate in phenol free medium containing the supplements as described above. Cells were grown for 48 hours and prior to treatment, cells were washed with 1× PBS and replaced with fresh phenol-free medium. To measure the change in extracellular O_2_^.−^ generated by XO/HX, XTT (100 µm) was added to the cells along with either XO/HX or vehicle. The change in absorbance at 470 nm was read by a Biorad 680 microplate reader (Biorad, Hercules, CA) at 1 hr following XO/HX. One set of cells were pre-treated for 30 minutes with 500 U/mL SOD (Sigma) prior to the addition of XO/HX. The experiment was repeated three times. Using the extinction coefficient of 21600 M^−1^ cm^−1^ (XTT), the superoxide flux was calculated and data expressed as rate of superoxide flux (M•s^−1^). Hydrogen peroxide was measured in calf PASMC treated with 1 hour of XO/HX using a fluorometric assay as described by Hyslop *et al* [24] and the rate of H_2_O_2_ production was expressed in M•s^−1^. Briefly, in this assay, cells are rinsed and incubated with phenol red-free buffer containing horseradish peroxidase (HRP, 1.6 mM), HEPES buffer, NaHCO_3_ (60 mM) and *para*-hydroxyphenyl acetic acid (*p*HPA, 95 µg/mL) for two hours. The reaction of H_2_O_2_ with HRP forms compound I, which then oxidizes *p*HPA and results in the formation of a fluorescent dimer product detectable by a fluorometric plate reader (polarstar Omega, BMG Labtech) [24].

### SOD Activity assay

Pulverized lung and pulmonary artery tissue (300 mg) were homogenized in 10 volumes of ice-cold buffer (50 mM potassium phosphate, pH 7.4, with 0.3 M KBr, 0.05 mM phenylmethylsulfonyl flouride, and 3 mM diethylene-triaminepentaacetic acid) and centrifuged to remove cellular debris. For the pulmonary artery homogenates, EC-SOD was separated from intracellular SOD (Cu,Zn SOD and Mn SOD) using a concanavalin A column (Pierce,Rockford,Illinois) as previously described [25]. Briefly, pulmonary artery homogenates were applied to the column. Intracellular SODs were collected by washing the column with 5 mL of equilibration buffer (50 mM HEPES, pH 7.0 with 0.25 M NaCl). The EC-SOD fraction was eluted with 3 mL 50 mM HEPES, pH 7.0 with 0.25 M NaCl with 1 M alpha-methylmannoside, final pH 5. The EC-SOD fraction was concentrated 10-fold by centrifuging with a centriprep concentrator (Amicon-ultracel-10K, Millipore) at 4°C for 60 minutes. EC-SOD and intracellular SOD separation was confirmed by Western blot, as described above, and activity levels were measured using the SOD assay kit-WST (Dojindo Molecular Technologies, Maryland, USA). This kit utilizes a water-soluble tetrazolium salt, WST-1 [2-(4-iodophenyl)-3-(4-nitrophenyl)-5-(2,4-disulfo-phenyl)-2*H*-tetrazolium, monosodium salt], to produce a water-soluble formazan dye upon reduction with a superoxide anion, detectable by a colorimetric assay. A standard curve was linear between SOD concentrations of 0.1 to 5 U/mL. SOD activity data were expressed as units of SOD activity per gram of tissue.

### GSH/GSSG assay

The GSH/GSSG ratio was measured as a marker of oxidative stress in PA tissue following chronic hypoxia as previously described [26], [27]. Briefly, cow PA tissue (50 mg) was homogenized in MES (Morpholino ethanesulfonic acid) (2∶1, w/v) at 4°C and centrifuged at 12,000 g. Total glutathione was determined after reducing GSSG to GSH with glutathione reductase by the method of Anderson [26]. GSSG alone was determined by incubating the sample with 2-vinyl pyridine to eliminate the GSH. The glutathione concentrations were normalized to the protein content in the sample as assayed by the Pierce 660 nm protein assay reagent (Thermo Scientific) and the ratio of GSH/GSSG was calculated.

### Cell Proliferation and Apoptosis Assays

These assays were carried out using human PASMC. Cell proliferation was measured by MTS assay using CellTiter 96 AQueous One Solution (Promega, Madison, WI). Twenty-dour hours after transfection with siRNA, ∼2000 cells were plated in a 96-well plate and twenty microliters of 3-(4,5-dimethylthiazol-2-yl)-5-(3-carboxymethoxyphenyl)-2-(4-sulfophenyl)-2H-tetrazolium inner salt (MTS) was added to the wells on consecutive dates. MTS is bio-reduced by cells into a colored formazan product that reduces absorbance at 490 nm. Plates were read at 24 and 48 hours using a BioTek *MODEL* plate reader (Winooski, VT) two hours after MTS reagent was added. Apoptosis was assayed forty-eight hours after siRNA transfection. Phosphatidylserine externalization (a marker of early apoptosis) was analyzed using the Guava Nexin reagent. Cells were counted following staining with Guava ViaCount reagent (Millipore, Billerica, MA) and the amount of apoptosis determined using Guava Nexin reagent (Millipore). Samples were run on a Guava EasyCyte Plus flow cytometer (Millipore). Experiments were done in triplicate.

### Statistical Analysis

Data are expressed as means ± SE. Unpaired t-test analysis or one-way Anova with Bonferroni post-hoc analysis was performed using Prism software (GraphPad, San Diego, CA, USA). Statistical significance was defined as *p*<0.05.

## Results

### A. From experiments with *in vivo* tissue samples

#### Chronic hypoxia decreases EC-SOD activity in the pulmonary artery of the neonatal calf

**Figure 1:**
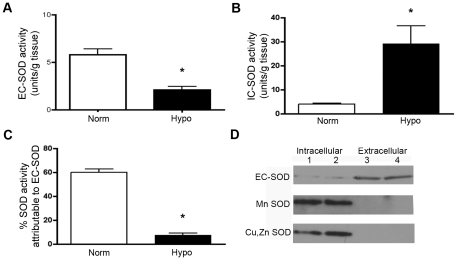
EC-SOD is decreased in the pulmonary artery in response to chronic hypoxia. In pulmonary artery tissue homogenates from two-week old chronically hypoxic (Hypo) and age-matched normoxic (Norm) control calves, extracellular SOD was separated from intracellular SODs (Cu,Zn SOD or SOD1 and Mn SOD or SOD2) using a concavalin A sepharose column, based on EC-SOD's predominantly glycosylated state that enables it to bind to the column and thus separate intracellular SODs (nonbound) from extracellular SOD (bound, then eluted) fraction. SOD activity was measured in each fraction using the SOD assay kit-WST. **A.** EC-SOD activity. **B.** Intracellular SOD (IC-SOD) activity. **C.** The percent of total SOD activity attributable to EC-SOD. Data expressed as mean ± SEM. *p<0.05 vs. Norm; n = 5. **D.** Western blot analysis of Cu,Zn SOD, Mn SOD and EC-SOD in the intracellular and extracellular fractions to confirm adequate separation of IC-SOD from EC-SOD. Lane 1 and 2 are the intracellular fractions from the two normoxic calves and the corresponding extracellular fraction is shown in Lanes 3 and 4.

We speculated that chronic hypoxia would lead to a loss of EC-SOD activity in the pulmonary artery of the chronically hypoxic neonatal calf, similar to our observation in the lungs of weanling mice exposed to hypoxia [10]. We separated the intracellular SODs from extracellular SOD in bovine pulmonary artery homogenates using a concavalin A column and measured the activity in each fraction. Extracellular SOD activity in the pulmonary artery was decreased by 36% following 14 days of chronic hypoxia compared to normoxic sample (**Figure 1A**). In contrast, the intracellular SOD activity levels increased 7.6-fold (**Figure 1B**). Furthermore, while extracellular SOD comprised 60% of total SOD activity under normoxic conditions, it only accounted for 7.5% of total SOD activity by the end of the hypoxic exposure (**Figure 1C**). We performed Western blot analysis on the two fractions of SOD to confirm that the Cu,Zn SOD (SOD1) and Mn SOD (SOD2) were only detectable in the intracellular fraction (IC-SOD), while EC-SOD (SOD3) was almost exclusively bound to the concavalin A column based on its glycosylated state, and eluted in the extracellular SOD fraction. (**Figure 1D**) Further, we performed several comparison measurements of SOD activity and EC-SOD expression in the mouse and calf lung tissue to corroborate the low enzymatic defenses against extracellular O_2_^.−^ measured in the neonatal calf. We observed the total SOD activity was significantly lower in two week old calf lungs compared with immature (four week old) mouse lungs (**Figure S1A**). Furthermore, when we performed Western blot analysis using 25–100 µg protein prepared from peripheral calf lung or pulmonary arteries, we were unable to detect any signal with an EC-SOD antibody. We have previously observed that EC-SOD is easily detectable in 25 µg mouse lung, and we used 1 µg purified bovine EC-SOD (kindly provided by Dr. Tim Oury, University of Pittsburgh.) as a positive control (**Figure S1B**). Therefore, any small change in SOD activity level in calf is crucial as it is more prone to oxidative stress. Despite the increase in intracellular SOD, there was no significant change in Cu,Zn or Mn SOD protein expression (**Figure S1C**). As there was a significant decrease in EC-SOD in calf PA after chronic hypoxia, we carried out further experiments with calf PA to study the role of ECSOD and the impact of chronic hypoxia.

#### Oxidative stress is increased in the pulmonary artery of chronically hypoxic neonatal calves

**Figure 2:**
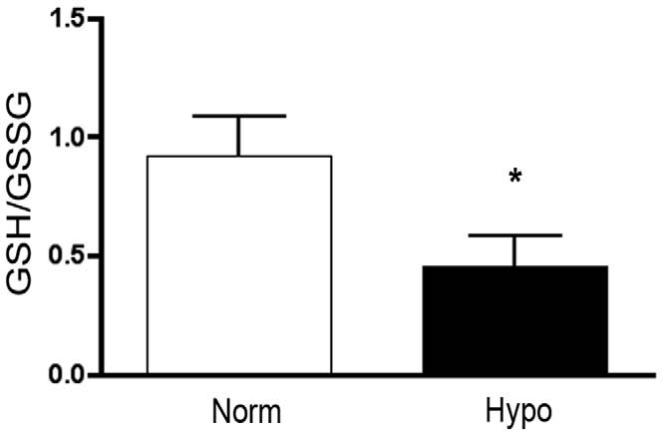
The GSH/GSSG ratio is decreased in the pulmonary artery of the chronic hypoxic calf (Hypo) compared to the normoxic control calf (Norm). **A.** GSH and total glutathione levels were measured and data expressed as the ratio of GSH to GSSG. Data expressed as mean ± SEM. *p<0.05 vs. Norm; n = 4.

Since EC-SOD activity is markedly decreased in the tissues of chronic hypoxic calves, it could trigger oxidative stress. Oxidative stress in the pulmonary artery was evaluated by determining the ratio of reduced to oxidized glutathione (GSH/GSSG) in the calf PA following 2 weeks of hypoxia. During periods of increased oxidative stress, GSSG will accumulate and the ratio of GSH to GSSG will decrease. Therefore, the determination of the GSH/GSSG ratio is a general indicator of oxidative stress in cells and tissues. We observed that the ratio of GSH/GSSG in calf PA decreased by 50% with hypoxia (**Figure 2**). The ratio was low in calf PA compared to our previously published findings in the mouse lung [10].

#### The redox sensitive transcription factor, Egr-1, is increased in the pulmonary artery of the chronically hypoxic neonatal calf

**Figure 3:**
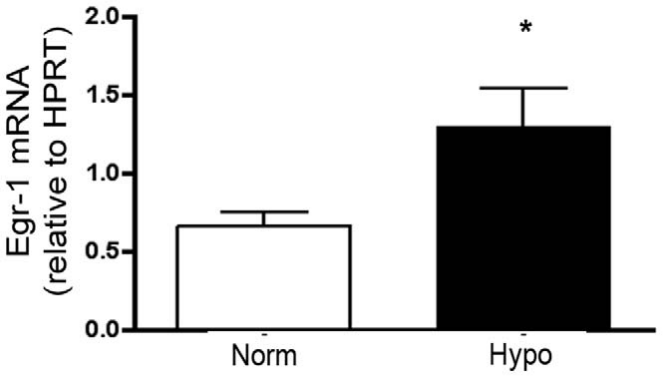
The redox-sensitive transcription factor Egr-1 is increased in the pulmonary artery of chronically hypoxic calves (Hypo) compared to age-matched normoxic control calves (Norm). Egr-1 mRNA expression was measured by qPCR in the pulmonary artery of 2 week old chronically hypoxic calves compared to age-matched normoxic control calves . Data are expressed as copies of Egr-1 per copy HPRT, mean ± SEM. *p<0.05 vs. Norm; n = 4–5.

We have previously reported that the level of EC-SOD activity in the lung modulates the hypoxic upregulation of Egr-1 in mice [10]. In addition, it has previously been reported that Egr-1 is highly expressed in the pulmonary artery adventitia of chronically hypoxic calves and Egr-1 contributes to hypoxia-induced cyclin D1 expression and fibroblast proliferation [10]. To confirm the vascular changes in Egr-1, we compared the mRNA expression of Egr-1 in lung and intraparenchymal proximal pulmonary artery of chronically hypoxic calves compared to age-matched normoxic calves. We found that Egr-1 mRNA expression did not increase in the lung tissue (data not shown) but was increased by 2 fold in the pulmonary artery of chronically hypoxic calves (**Figure 3**).

### B. From *in vitro* experiments with calf PASMC cells

#### Extracellular reactive oxygen species upregulate Egr-1 in pulmonary artery smooth muscle cells (PASMC)

**Figure 4:**
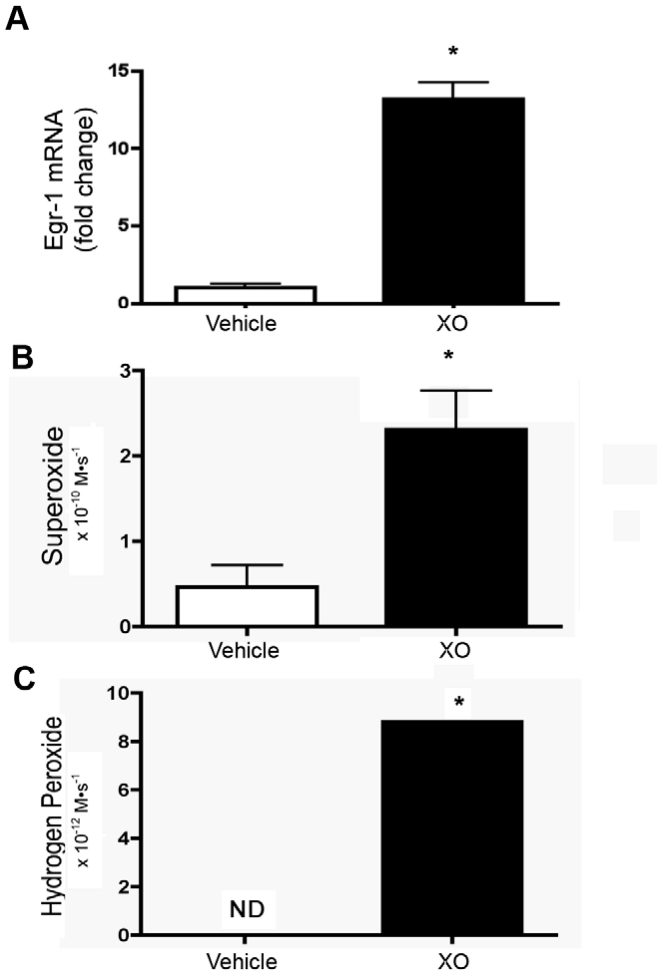
Xanthine oxidase upregulates Egr-1 in calf pulmonary artery smooth muscle cells. (**A**) Egr-1 mRNA expression was measured by qPCR in PASMC exposed to xanthine oxidase (XO- 8 mU/mL) and hypoxanthine (0.5 mM) at 37°C for 1 hour (XO). Data are expressed as fold change in Egr-1/HPRT relative to vehicle treated cells and presented as mean ± SEM. *p<0.05 vs. vehicle treated cells. **B.** Extracellular O_2_^.−^ was measured by the SOD-inhibitable reduction of XTT in XO+hypoxanthine-treated (XO), and vehicle-treated smooth muscle cells with and without SOD (500 U/mL). 20,000 cells were treated in a final volume of 150 µL and exposed to XTT for 1 hour at 37°C and pH 7.4. An extinction coefficient of 21600 M^−1^ cm^−1^ (XTT) was used to calculate O_2_^.−^ flux and data expressed in M•s^−1^. *p<0.05 vs. vehicle-treated cells, n = 3. **C.** H_2_O_2_ was measured with a standard fluorometric assay utilizing in which H_2_O_2_ reacts with HRP to forms compound I, which subsequently oxidizes *p*HPA to its dimer form which can be detected on a fluorometer. Experiments were performed with 30,000 cells per well and a final volume of 150 µL for 2 hours at 37°C and pH 7.4 and H_2_O_2_ flux expressed in M•s^−1^. *p<0.05 vs. vehicle-treated cells, n = 3. Data expressed as mean ± SEM.

To evaluate whether the increased Egr-1 expression detected in the pulmonary artery of chronically hypoxic calves could be directly mediated by extracellular ROS, we performed a series of experiments in SMC treated with XO+HX as an exogenous source of ROS. A dose response curve with a concentration of XO between 1–16 mU/mL and a time course between 30 minutes to 4 hours was initially performed to select the experimental conditions (**Figure S2**). Based on these pilot studies, we selected one hour incubation with 8 mU/mL XO and 0.5 mM HX for subsequent experiments. This experimental condition significantly increased Egr-1 mRNA expression in SMC (**Figure 4A**).

**Figure 5:**
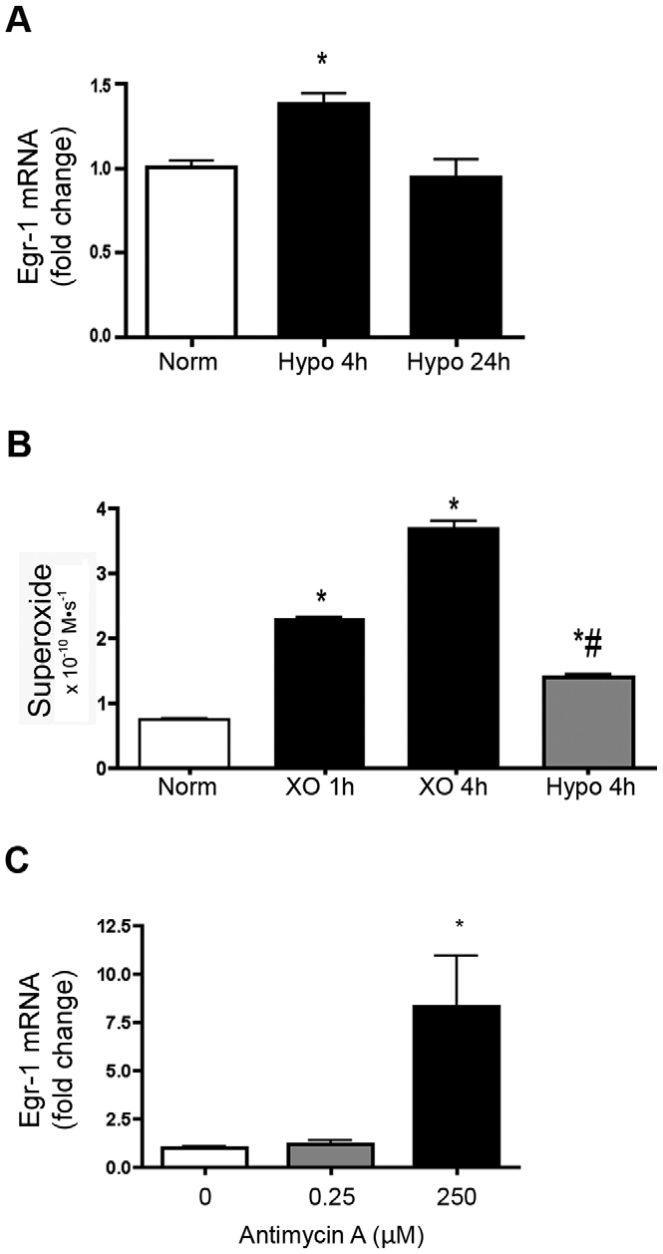
Exposure of calf PASMC to hypoxia resulted in less induction of Egr-1 and less extracellular superoxide generation than XO. **A.** Egr-1 mRNA expression in calf PASMC exposed in sealed humidified gas chambers with 21% or 1% O_2_ plus 5% CO_2_ and balanced N_2_ at 37°C for 4 and 24 hours and shown compared to XO/HX treatment for 1 hour (XO). **B.** Extracellular O_2_^.−^ production in PASMC exposed to XO/HX (XO) or hypoxia as described above. 20,000 cells treated in a final volume of 150 µL were exposed to XO/HX (XO) for 1 or 4 hours or hypoxia for 4 hours at 37°C and pH 7.4 in the presence of XTT. An extinction coefficient of 21600 M^−1^ cm^−1^ was used to calculate O_2_^.−^ flux (M-s^−1^). **C.** Egr-1 mRNA expression in calf PASMC exposed to antimycin A to increase endogenous ROS generated in the mitochondria. 20,000 cells were exposed to 0.25 µM or 250 µM antimycin A for 2 hours at 37°C. All data expressed as mean ± SEM. *p<0.05 vs. vehicle-treated cells, n = 3.

XO generates extracellular O_2_^.−^, which rapidly dismutates to H_2_O_2_, though XO can also generate H_2_O_2_ directly, depending on conditions such as pH or oxygen concentration [28]–[30]. In our experimental conditions, we detected an increase in extracellular O_2_^.−^ flux when cells were treated with XO/HX for 1 hour, as shown by the SOD-inhibitable reduction of XTT, which specifically reflects extracellular O_2_^.−^ generation (**Figure 4B**). H_2_O_2_ production was also significantly elevated following a 1 hour treatment with XO/HX while H_2_O_2_ was below the level of detection in vehicle-treated cells, though the rate of accumulation of H_2_O_2_ was lower than the production of O_2_^.−^ indicating that extracellular H_2_O_2_ was being rapidly removed by the cells, likely through GSH (**Figure 4C**). Compared to PASMC treated with XO/HX, exposure of cells to hypoxia produced a significantly smaller rise in Egr-1 mRNA (**Figure 5A**) as well as less extracellular O_2_^.−^ production (**Figure 5B**). Low dose antimycin A, at a dose known to generate endogenous ROS due to inhibition of complex III of the mitochondrial electron transport chain, did not upregulate Egr-1, though high dose antimycin A did result in an increase in Egr-1 mRNA (**Figure 5C**).

#### Xanthine oxidase-derived extracellular reactive oxygen species upregulated Egr-1 via the activation of ERK1/2 in pulmonary artery smooth muscle cells

**Figure 6:**
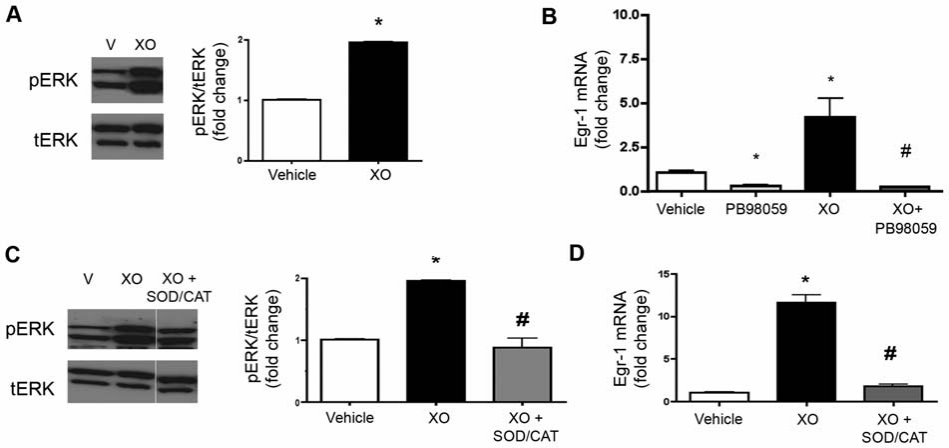
ROS derived from xanthine oxidase increase Egr-1 expression through ERK1/2 signaling. Calf PASMC were treated with XO (8 mU/mL) and hypoxanthine (0.5 mM) for 1 hour (XO) and protein was isolated for Western blot analysis. **A.** Blots were probed with antibodies against phosphorylated ERK1/2 (pERK) and total ERK1/2 (tERK) Each panel shows representative lanes from a single immunoblot as well as the densitometry data representing the ratio of the phosphorylated to total ERK1/2. **B.** Calf PASMC were pretreated with the MAPK/ERK1/2 inhibitor PD98059 (10 µM) for 30 min followed by either XO/HX (XO) or vehicle control. mRNA was isolated and analyzed by qPCR for Egr-1 expression relative to HPRT. **C.** Calf PASMC were pretreated with CuZn SOD (500 U/mL) and catalase (600 U/mL) for 30 minutes prior to XO (8 mU/mL) and hypoxanthine (0.5 mM). Total protein was subjected to Western blot analysis for phosphorylated ERK1/2 (pERK) and total ERK1/2 (tERK). The band intensities were quantified by densitometry and normalized to that of vehicle. **D.** PASMC were pretreated with CuZn SOD (500 U/mL) and catalase (600 U/mL) for 30 minutes prior to XO (8 mU/mL) and hypoxanthine (0.5 mM). mRNA was isolated and analyzed by qPCR for Egr-1 expression relative to HPRT. All data expressed as mean ± SEM. *p<0.05 vs. vehicle treated cells; ^#^p<0.05 vs. 1 hour XO-treated cells; n = 3.

**Figure 7:**
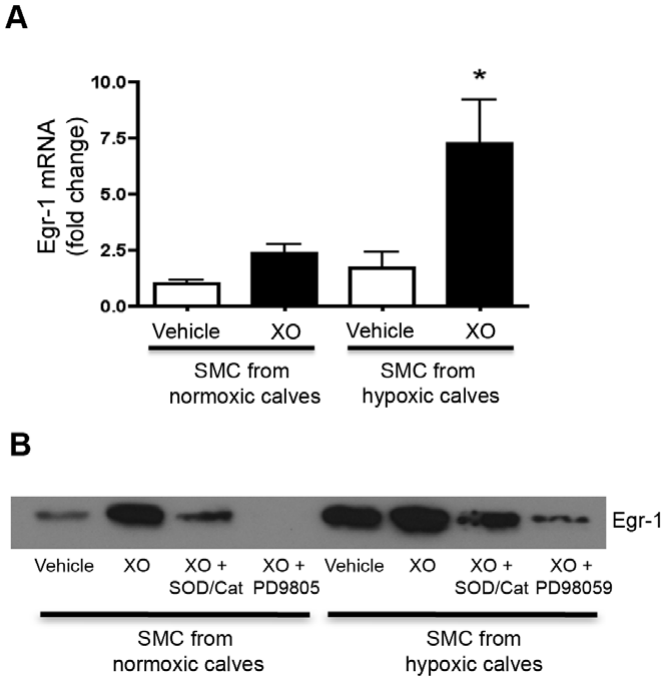
PASMC isolated from chronically hypoxic calves had a more marked upregulation of Egr-1 in response to xanthine oxidase compared to cells isolated from normoxic calves. **A.** PASMC isolated from three chronically hypoxic calves and three normoxic age-matched control calves were treated simultaneously with xanthine oxidase (8 mU/mL) and hypoxanthine (0.5 mM) at 37°C for 1 hour (XO). Egr-1 mRNA expression was measured by qPCR and expressed as fold change in Egr-1/HPRT relative to the vehicle-treated PASMC isolated from normoxic calves. Data are presented as mean ± SEM. *p<0.05 vs. vehicle treated cells. **B.** For protein analysis, PASMC (1×10^6^ cells per plate) isolated from a chronically hypoxic calf and a normoxic age-matched control calf were treated simultaneously with xanthine oxidase (8 mU/mL) and hypoxanthine (0.5 mM) at 37°C for 1 hour (XO) with and without a 30 minute pretreatment with either Cu,Zn SOD (500 U/mL) and catalase (600 U/mL) or with the MAPK/ERK1/2 inhibitor PD98059 (10 µM). Nuclear protein was isolated using Thermo-Scientific NE-PER Nuclear and Cytoplasmic Extraction Reagents, and 25 µg was loaded on a gel for Western blot analysis with the Egr-1 antibody (1∶1,000). Ponceau S staining was performed on the gel to confirm protein equal loading. (see Figure S4).

MAPK/ERK signaling pathways have been shown to regulate Egr-1, and many MAPK enzymes are redox sensitive. To determine if extracellular O_2_^.−^, or H_2_O_2_ arising from the dismutation of O_2_^.−^, induced the upregulation of Egr-1 via MAPK, we first evaluated whether XO/HX treatment resulted in phosphorylation of ERK1/2. Treatment with XO/HX led to phosphorylation of ERK1/2 (**Figure 6A**) in PASMC. Pretreatment with the ERK1/2 inhibitor, PD98059 (10 µM) attenuated the XO/HX-mediated upregulation of Egr-1, as measured by qPCR (**Figure 6B**). We also detected an increase in Egr-1 protein levels in the nuclear protein of cells exposed to XO/HX that was blocked by the PD98059. (**Figure 7B**). The combined pretreatment of cells with SOD and catalase reversed both XO/HX-mediated ERK1/2 phosphorylation (**Figure 6C**) as well as upregulation of Egr-1 mRNA and protein (**Figure 6D** **and** **7B**). Catalase also significantly attenuated the upregulation of Egr-1 by XO/HX, while pretreatment with SOD alone did not prevent the upregulation of Egr-1 (**Figure S3**). These data cumulatively demonstrate a loss of EC-SOD and increased oxidative stress in the PA of chronically hypoxic calves and define a role for extracellular ROS in the upregulation of Egr-1 via the activation of EKR1/2 signaling in the pulmonary circulation.

#### Xanthine oxidase treatment of PASMC isolated from chronically hypoxic calves with lower PA EC-SOD activity augmented the upregulation of Egr-1 mRNA and protein

Upon completing studies that tested the impact of extracellular O_2_^.−^ on Egr-1 expression, we performed a final series of experiments in PASMC isolated from normoxic and chronically hypoxic calves and in human PASMC following siRNA silencing of EC-SOD. These experiments were designed to confirm the impact of the level of EC-SOD expression on Egr-1 expression in the *in vitro* model and tested the impact of EC-SOD activity on SMC growth characteristics. The PASMCs isolated from chronically hypoxic calves showed a more significant increase in Egr-1 mRNA in response to XO/HX (**Figure 7A**) compared to cells isolated from normoxic calves. PASMC from three normoxic calves and three hypoxic calves were simultaneously passaged and treated with XO/HX to minimize experimental variability. Cells from both normoxic and hypoxic calves increased nuclear Egr-1 protein expression following XO/HX, with a higher signal detectable in cells isolated from a chronically hypoxic calf compared to cells isolated from a normoxic calf. Pretreatment with SOD+catalase or ERK inhibition attenuated the increase in XO/HX induced Egr-1 protein expression (**Figure 7B**). Equal loading in the nuclear extracts was confirmed via Ponceau S stain (**Figure S4**).

### C. From *in vitro* experiments with human PASMC cells

#### EC-SOD silencing in human PASMC by siRNA transfection augmented Egr-1 mRNA and protein expression

**Figure 8:**
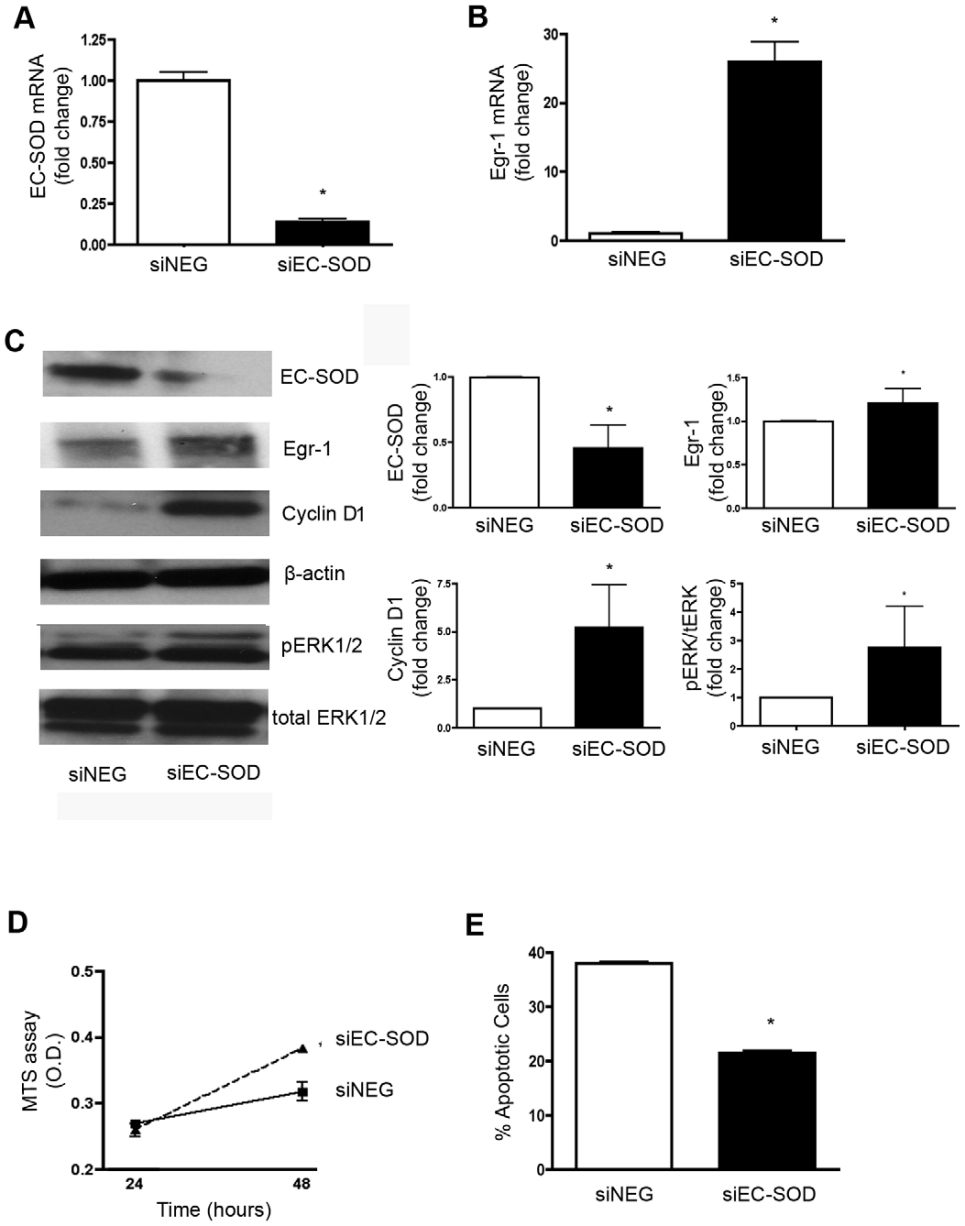
siRNA-mediated knock down of EC-SOD in human pulmonary artery smooth muscle cells increased Egr-1 mRNA and protein and promoted a pro-proliferative and anti-apoptotic phenotype. Human PASMC were transfected with human EC-SOD siRNA (siEC-SOD) or the negative control siRNA vector (siNEG) and experiments were performed 48 hours later. **A.** EC-SOD mRNA expression expressed relative to GAPDH to test the extent of knock-down of EC-SOD with siRNA. **B.** Fold change in Egr-1 mRNA expression expressed relative to GAPDH in human PASMC treated with siEC-SOD compared to cells treated with siNEG. Data are presented as mean ± SEM. *p<0.05 vs. siNEG. **C.** Western blot analysis in total cell lysates from human PASMC treated with siEC-SOD or siNEG. The blots were probed sequentially with the human EC-SOD antibody (1∶1,000), Egr-1 antibody (1∶500) cyclin D1 and ß-actin (1∶1,000) to confirm equal protein loading. Blots were also probed with the phosphorylated ERK1/2 and total ERK1/2 antibodies (1∶1,000). Densitometry shows fold change in EC-SOD, Egr-1 or cyclin D1 relative to ß-actin and fold change in phosphorylated relative to total ERK1/2 (n = 2). **D.** Cell proliferation measured by MTS assay. Experiments were done in triplicate. *p<0.05 vs. siNEG. Solid line (▪): siNEG; dashed line (▴): siEC-SOD *p<0.05 vs. siNEG **E**. Apoptosis was assayed using the Guava Nexin kit (Guava Technologies) according to the manufacturer's protocol. Fluorescence emission for Annexin V staining and 7-AAD was measured by flow cytometry. Experiments were done in triplicate. *p<0.05 vs. siNEG.

We then performed additional experiments with human PASMC to confirm the impact of EC-SOD on Egr-1 expression. We selected human PASMC because these cells express high levels of EC-SOD at baseline, thus knock-down of EC-SOD would have a more significant impact than in the calf cells with low EC-SOD expression levels at baseline. Human PASMC were transfected with human EC-SOD siRNA (siEC-SOD) or the negative control siRNA vector (siNEG), and EC-SOD mRNA and protein was measured. We first confirmed that we significantly knocked down EC-SOD mRNA (**Figure 8A**) and protein (**Figure 8C**) in human PASMC with siRNA treatment. Knock down of EC-SOD in human PASMC increased Egr-1 mRNA and protein, as measured by qPCR and Western blotting respectively. (**Figure 8B and 8C**) Since activated ERK1/2 is implicated in Egr-1 expression, and Egr-1 can promote cell proliferation by increasing cyclin D1, we measured phosphorylated and total ERK1/2 as well as cyclin D1 protein expression. Knock down of EC-SOD resulted in increased phosphorylated ERK1/2/total ERK1/2 and increased cyclin D1 protein levels (**Figure 8C**).

#### EC-SOD silencing in hPASMC by siRNA transfection led to increased cell proliferation and attenuated cell apoptosis

Pulmonary vascular cells isolated from humans with PAH or animals with experimental PH exhibit increased cell proliferation and decreased cell apoptosis. To evaluate the impact of EC-SOD on PASMC growth characteristics, we measured cell proliferation and apoptotic index following knock down of EC-SOD. After siRNA treatment to knock down EC-SOD, there was significant increase in cell proliferation (**Figure 8D**). We then measured the apoptotic status of cells after knock down of EC-SOD by flow cytometry. As shown in **Figure 8E**, inhibition of EC-SOD led to a significant decrease in the cell apoptotic index. These indicate that depletion of EC-SOD in human PASMC results in increased cell survival and proliferation.

## Discussion

Understanding the molecular mechanism that induces pulmonary hypertension in neonates is important for the development of improved therapeutics. In this current study, we used the chronically hypoxic calf model of pulmonary hypertension to evaluate the activity of EC-SOD in a severe neonatal disease model and to better understand the role for extracellular O_2_^.−^ in the upregulation of Egr-1, a redox sensitive transcription factor implicated in vascular remodeling. We tested the hypothesis that loss of EC-SOD in chronically hypoxic calves leads to extracellular O_2_^.−^ -mediated upregulation of Egr-1 via activation of MAPK pathways. We tested tissue from chronically hypoxic calves to demonstrate the decrease in EC-SOD in the pulmonary artery *in vivo* and then treated isolated pulmonary vascular smooth muscle cells with xanthine oxidase to show that ROS generated outside the cell could upregulate Egr-1 via activation of MAPK/ ERK1/2. We used human PASMC following siRNA knock-down of EC-SOD to confirm the impact of EC-SOD expression on Egr-1 expression and SMC proliferative ability. Our data provide new insight into the importance and targets of EC-SOD and extracellular O_2_^.−^ in chronic hypoxic pulmonary hypertension.

We previously reported that EC-SOD activity is impaired following exposure to chronic hypoxia, and overexpression of lung EC-SOD in two murine models of pulmonary hypertension protected against pulmonary vascular remodeling and prevented the early upregulation of the redox sensitive transcription factor, Egr-1 [10], [11]. In this study, we report that total SOD activity levels in the calf lung and pulmonary artery were very low compared to the mouse lung, and extracellular SOD activity decreased in the pulmonary artery of calves exposed to 2 weeks of chronic hypoxia. The low SOD activity level measured in the calf pulmonary artery tissue was also substantially less than published activity levels reported for baboon and human pulmonary artery; though we report, similar to the baboon and human, that EC-SOD activity accounted for the majority of the total SOD activity in the pulmonary artery under normal conditions [31]. The low activity levels of total SOD may be in part due to the young age of the calves, as lung antioxidant defenses are known to be low early in life and increase in the perinatal period to prepare for the relative hyperoxia of room-air breathing. Consistent with this, we previously reported in the developing rabbit that EC-SOD activity in the lung increased during the first month of life [25]. Several pieces of data support the premise that the low SOD activity is associated with an increase in oxidative stress in the pulmonary artery of the chronically hypoxic calf. The ratio of GSH/GSSG was low in the calf pulmonary artery and decreased further with hypoxia. This result is similar to our published observation in mice in which the GSH/GSSG ratio decreased by 50% in response to hypoxia [10]. In chronic hypoxic mice, where we were able to examine a time-course, we had reported an early increase in EC-SOD expression and activity, which may reflect an adaptive response to combat increased oxidant stress [22]. Interestingly, in this study, we measured an increase in intracellular SOD (IC-SOD) activity in the calf PA at 2 weeks of hypoxia. This may also be adaptive in response to increased intracellular oxidative stress. It is possible that intracellular SOD activity would decrease over time, as we observed in the lung of chronically hypoxic mice over a 5 week period, but the large animal models are limited by cost and the longer exposures were not feasible. The loss of EC-SOD activity was even more pronounced when considered as the percentage of total SOD activity, decreasing its contribution to total SOD activity from 60% to less than 10%. We speculate that an increased susceptibility to oxidative stress due to the low total SOD activity along with a further loss of extracellular EC-SOD could contribute to the significant remodeling in the medial and adventitial layer of the pulmonary arteries observed in the neonatal calf compared to the modest vascular remodeling characteristic of chronically hypoxic mice [19], [32]–[35].

To test the impact of extracellular O_2_^.−^on Egr-1 expression in PASMC, we treated cells with XO, as an enzymatic source of O_2_^.−^. This exogenous model has potential relevance to the *in vivo* setting, as XO is upregulated in the hypoxic pulmonary circulation and can contribute to pulmonary hypertension [4], [36]–[38]. A key finding in the cell culture experiments was that exogenous generation of extracellular O_2_^.−^ by XO in smooth muscle cells strongly upregulated Egr-1 mRNA and protein expression. Furthermore, cells isolated from chronically hypoxic calves had a more marked response to XO than cells isolated from normoxic calves. Since the SMC are a major source of vascular EC-SOD and we detected less EC-SOD in the pulmonary artery of chronically hypoxic calves, we speculate that the loss of EC-SOD in these cells contributed to the enhanced upregulation of Egr-1 in response to XO. This was supported by our finding in human PASMC in which knock-down of EC-SOD increased Egr-1 mRNA and protein. In the bovine cell culture model, pretreatment with both SOD and catalase was required to fully block the XO-induced signal. This indicates that H_2_O_2_, which also will increase with XO treatment due to the rapid dismutation of O_2_^.−^ or its direct generation of H_2_O_2_, was capable of inducing Egr-1 expression in this system. In contrast, in the human cells, knocked down of EC-SOD expression was sufficient to increase Egr-1, consistent with our previous observation in the mouse model, that overexpression of EC-SOD in the lung blunted the hypoxic induction of Egr-1 expression[10]. These data demonstrate that we can use XO as a model to test how extracellular ROS can regulate Egr-1 mRNA expression; however, we must also consider the impact of exogenous vs. endogenous sources of ROS and the model when we interpret the data. Our data support the conclusion that extracellular O_2_^.−^ may upregulate Egr-1 directly or following its dismutation to H_2_O_2_. Consistent with our cell culture findings, one published study reported that the exogenous administration of H_2_O_2_ in cardiac cells also increased Egr-1 mRNA and protein expression [39]. In our study when SOD+catalase was compared to catalase pretreatment alone, there is a small difference, suggesting that O_2_^.−^ may have an effect independent of its dismutation to H_2_O_2_. It is also possible that EC-SOD modulates Egr-1 expression by regulating NO bioavailability. This mechanism was not tested in this study. Overall, these data indicate that while the bovine cell culture experiments do not fully mimic the *in vivo* setting, extracellular O_2_^.−^, either directly or indirectly following its dismutation to H_2_O_2_, can upregulate Egr-1 in smooth muscle cell, and provides an opportunity to further understand how O_2_^.−^ can regulate the redox sensitive transcription factor, Egr-1.

Published studies have shown enhanced induction of Egr-1 with hypoxia [10], [14]–[19], For example, fetal bovine pulmonary artery fibroblasts exhibited an increase in Egr-1 mRNA by Northern blot analysis following four hours of 3% oxygen [18]. However, we observed that exposure of PASMCs to hypoxia (1% for 1–4 hours), in addition to generating lower concentrations of extracellular O_2_^.−^ than XO, also had a much smaller impact on Egr-1 mRNA expression. This demonstrates that hypoxic induction of Egr-1 is specific to tissue or cell types, particularly given the known heterogeneity in cells within the vessel wall. The response can also vary with the hypoxic condition and the extent of hypoxic exposure. We speculate that an important source of extracellular ROS in the pulmonary circulation is that generated by recruited or resident inflammatory cells in response to hypoxia. Thus, a short bolus exposure to hypoxia in a single vascular cell type does not mimic the in vivo model system. While we focused in this study on the impact of extracellular O_2_^.−^ on Egr-1 regulation, our data with antimycin A suggest that under certain conditions, intracellular ROS production can also modulate the expression of this transcription factor. Further studies will dissect the origin and sources of extracellular ROS, distinct from cytosolic or mitochondrial sources, in the lung and pulmonary circulation in response to hypoxia. Since the use of hypoxia in cultured vascular cells was limited by the low levels of extracellular O_2_^.−^ generated and a minimal impact on Egr-1 regulation, we selected XO treatment as a model to test whether extracellular O_2_^.−^ upregulated Egr-1 via the activation of MAPK pathways.

There is extensive data implicating the MAPK/ERK1/2 pathway in the regulation of Egr-1 and in the pathogenesis of chronic hypoxic pulmonary hypertension [1], [17], [18], [39]–[41]. Furthermore, it is well-established that reactive oxygen species can activate MAPK through its phosphorylation [42]. These two observations formed the basis of our decision to test whether ROS generated outside the cell upregulated Egr-1 via MAPK. We found that extracellular ROS, generated by XO, phosphorylated ERK1/2, while inhibition of the ERK1/2 pathway prevented the upregulation of Egr-1. This identifies the importance of MAPK/ERK pathway in the redox regulation of Egr-1 in neonatal PASMC. A recent study showed that an increased extracellular oxidation in vascular smooth muscle cells activated the EGFR membrane receptor, leading to phosphorylation of ERK1/2 and activation of downstream transcription factors [43]. Our work thus provides a basis for future studies to better understand how the activity of EC-SOD may regulate extracellular redox state and further dissect its targets leading to ERK1/2 phosphorylation and regulation of Egr-1. Accordingly, targeting Egr-1 regulation may represent a novel therapeutic strategy to prevent PA remodeling.

The regulation of Egr-1 has been implicated in vascular remodeling in a number of models including the calf model of chronic hypoxic pulmonary hypertension. For example, upregulation of Egr-1 contributes to cyclin D1 expression and hypoxia-induced cell proliferation in fetal lung fibroblasts and stimulates insulin-like growth factor-1 receptor, resulting in vascular remodeling of vein grafts [44]. In addition, a pro-proliferative and anti-apoptotic phenotype has been attributed to SMC in pulmonary hypertension. Consistent with these studies, we were also able to show that knock-down of EC-SOD in human PASMC upregulated cyclin D1 and ERK1/2 activation, augmented proliferation and blunted apoptosis, providing direct evidence for EC-SOD in regulating PASMC phenotype.

In summary, we report that the neonatal calf has low SOD activity levels in the pulmonary artery and EC-SOD activity decreases further in the calf with pulmonary hypertension secondary to chronic hypoxia. The loss of EC-SOD is associated with an increase in Egr-1 mRNA in the pulmonary artery. We thus tested the impact of extracellular O_2_^.−^ and its product, H_2_O_2_, on Egr-1 expression in vascular smooth muscle and found that exogenous production of these ROS, via xanthine oxidase, upregulated Egr-1. Hypoxia itself had a minimal effect on either Egr-1 expression or O_2_^.−^ production. Extracellular ROS together with activation of ERK1/2 by phosphorylation regulated the increase in Egr-1. Therefore, targeting Egr-1 by controlling extracellular ROS generation and thereby balancing the cellular redox status could be a potential therapeutic pathway for pulmonary artery remodeling. Our data provided new insight into the role of extracellular O_2_^.−^ and EC-SOD in the pathogenesis of pulmonary hypertension.

## Supporting Information

Figure S1 **Total SOD activity is less in the calf lung compared to the mouse lung.** **A.** Total lung SOD, measured with an SOD activity kit (Dojindo Molecular Technologies) and expressed as units of SOD activity per gram tissue (U/g tissue), compared the activity in control two week old calves to immature control four week old mice. *p<0.05; n = 5. **B.** Western blot analysis of calf lung protein (25 µg) and purified bovine EC-SOD (1 µg) with a rabbit polyclonal EC-SOD antibody quantified. **C.** Representative Western blot of total PA homogenates for Cu,Zn SOD and Mn SOD with densitometry normalized to ß-actin, n = 5–6. p = 0.07 between groups for both blots.
(TIF)

Figure S2 **Dose response curve and time course for xanthine oxidase on Egr-1 mRNA expression.** **A.** Calf PASMC were treated with 0, 1, 4, 8 and 16 mU/mL xanthine oxidase+0.5 mM hypoxanthine for 1 hour and Egr-1 mRNA expression was determined by qPCR. Experiment was performed in triplicate and data expressed as Egr-1/HPRT relative to control cells. *p<0.05 vs control. **B.** Calf PASMC were treated with 8 mU/mL xanthine oxidase+0.5 mM hypoxanthine for 0.5, 1, 2 and 4 hours and Egr-1 mRNA expression was determined by qPCR. Experiment was performed in triplicate and data expressed as Egr-1/HPRT relative to vehicle-treated control cells. *p<0.05 vs control.
(TIF)

Figure S3 **Combined treatment with SOD and CAT significantly inhibited XO induced Egr-1 expression.** PASMC were pretreated with SOD (500 U/mL), Cat (600 U/mL) or combined SOD+CAT for 30 minutes prior to xanthine oxidase (8 mU/mL) and hypoxanthine (0.5 mM) to evaluate the contribution of superoxide and hydrogen peroxide to Egr-1 upregulation. mRNA was isolated analyzed by real-time RT-PCR for Egr-1 and HPRT expression. *p<0.05 vs. Vehicle and ^#^p<0.05 vs. XO+SOD treatment.
(TIF)

Figure S4 **Ponceau S stain of the blot presented in** **Figure 7** **to demonstrate equal protein loading.** The membrane shown in Figure 7B was stained with Ponceau S to confirm equal nuclear protein loading.
(TIF)
