# Supplementary figures and images for: Xanthine Oxidase-Derived ROS Upregulate Egr-1 via ERK1/2 in PA Smooth Muscle Cells; Model to Test Impact of Extracellular ROS in Chronic Hypoxia
Source: PLoS One. 2011 Nov 28;6(11):e27531. doi: 10.1371/journal.pone.0027531 (PMC3225357; doi:10.1371/journal.pone.0027531)

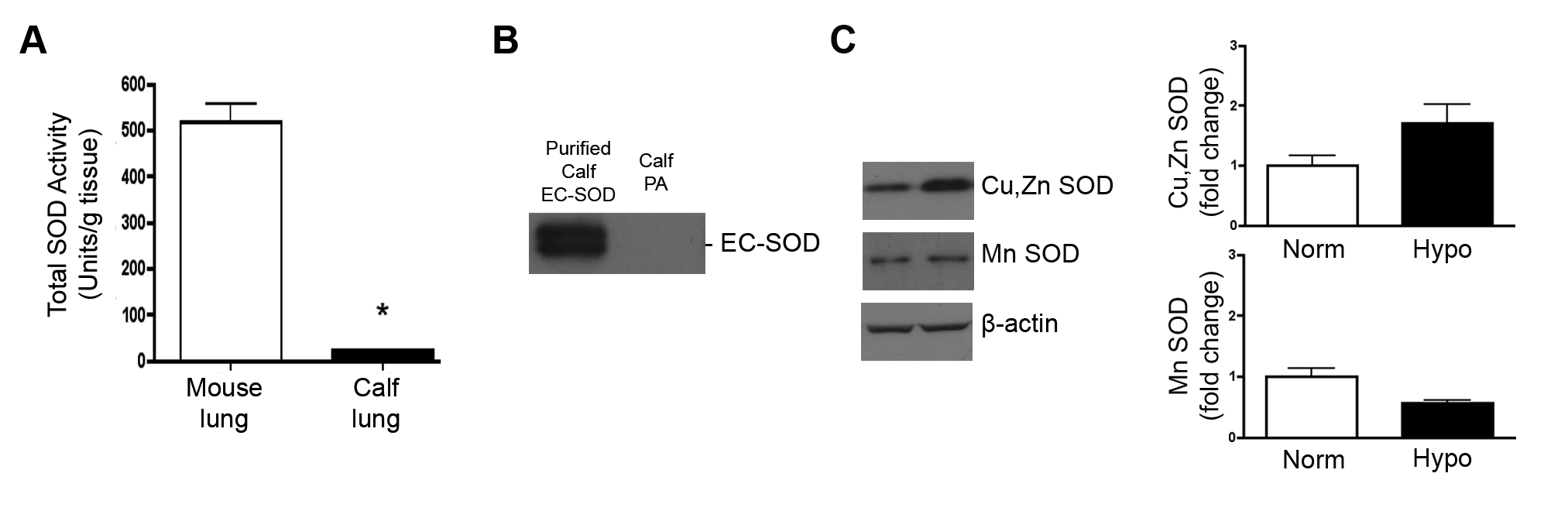

Supplement: Figure S1 — Total SOD activity is less in the calf lung compared to the mouse lung. A. Total lung SOD, measured with an SOD activity kit (Dojindo Molecular Technologies) and expressed as units of SOD activity per gram tissue (U/g tissue), compared the activity in control two week old calves to immature control four week old mice. *p<0.05; n = 5. B. Western blot analysis of calf lung protein (25 µg) and purified bovine EC-SOD (1 µg) with a rabbit polyclonal EC-SOD antibody quantified. C. Representative Western blot of total PA homogenates for Cu,Zn SOD and Mn SOD with densitometry normalized to ß-actin, n = 5–6. p = 0.07 between groups for both blots. (TIF) [file pone.0027531.s001.tif]

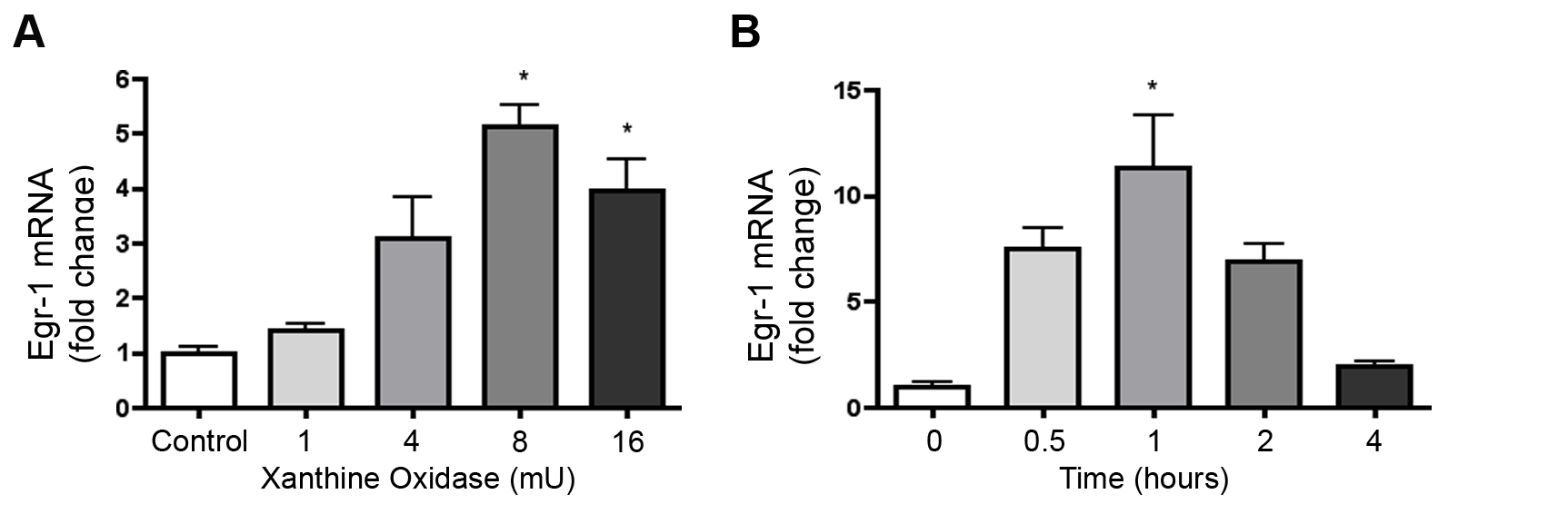

Supplement: Figure S2 — Dose response curve and time course for xanthine oxidase on Egr-1 mRNA expression. A. Calf PASMC were treated with 0, 1, 4, 8 and 16 mU/mL xanthine oxidase+0.5 mM hypoxanthine for 1 hour and Egr-1 mRNA expression was determined by qPCR. Experiment was performed in triplicate and data expressed as Egr-1/HPRT relative to control cells. *p<0.05 vs control. B. Calf PASMC were treated with 8 mU/mL xanthine oxidase+0.5 mM hypoxanthine for 0.5, 1, 2 and 4 hours and Egr-1 mRNA expression was determined by qPCR. Experiment was performed in triplicate and data expressed as Egr-1/HPRT relative to vehicle-treated control cells. *p<0.05 vs control. (TIF) [file pone.0027531.s002.tif]

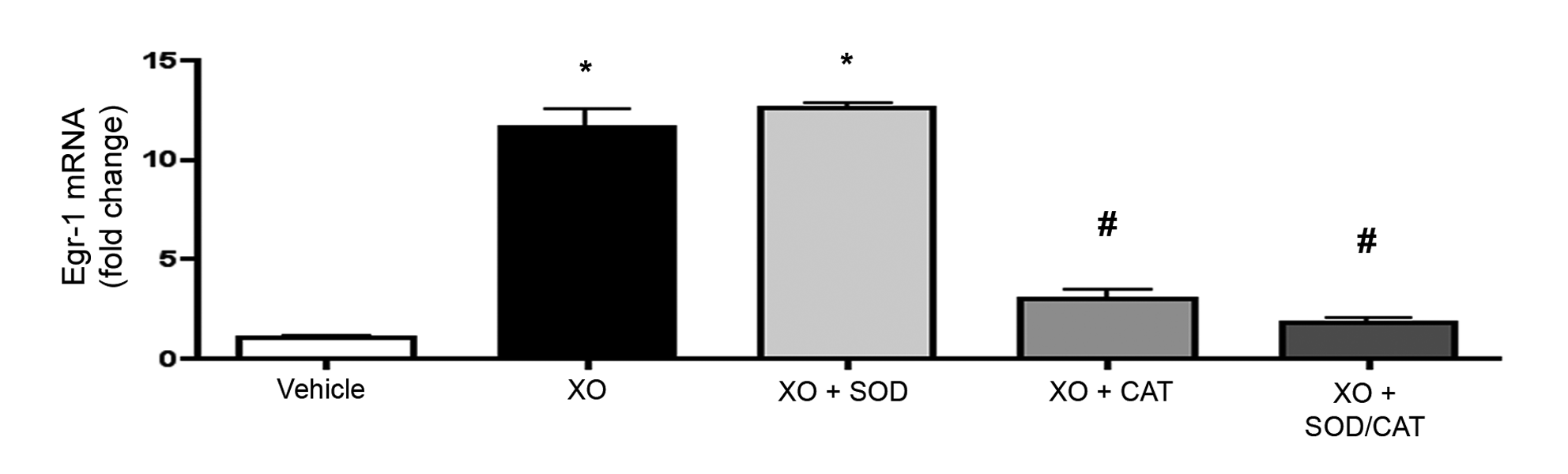

Supplement: Figure S3 — Combined treatment with SOD and CAT significantly inhibited XO induced Egr-1 expression. PASMC were pretreated with SOD (500 U/mL), Cat (600 U/mL) or combined SOD+CAT for 30 minutes prior to xanthine oxidase (8 mU/mL) and hypoxanthine (0.5 mM) to evaluate the contribution of superoxide and hydrogen peroxide to Egr-1 upregulation. mRNA was isolated analyzed by real-time RT-PCR for Egr-1 and HPRT expression. *p<0.05 vs. Vehicle and #p<0.05 vs. XO+SOD treatment. (TIF) [file pone.0027531.s003.tif]

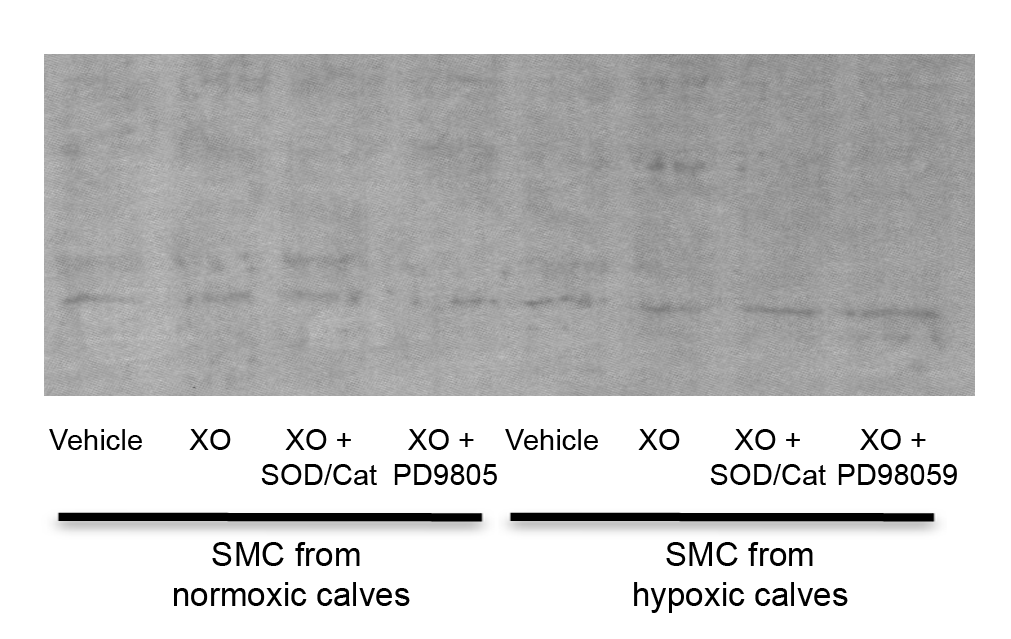

Supplement: Figure S4 — Ponceau S stain of the blot presented in Figure 7 to demonstrate equal protein loading. The membrane shown in Figure 7B was stained with Ponceau S to confirm equal nuclear protein loading. (TIF) [file pone.0027531.s004.tif]
